# Live and let die: epigenetic modifications of Survivin and Regucalcin in non-small cell lung cancer tissues contribute to malignancy

**DOI:** 10.1186/s13148-019-0770-6

**Published:** 2019-11-12

**Authors:** Dörte Nitschkowski, Sebastian Marwitz, Sousana A. Kotanidou, Martin Reck, Christian Kugler, Klaus F. Rabe, Ole Ammerpohl, Torsten Goldmann

**Affiliations:** 1 Pathology of the University Medical Center Schleswig-Holstein, Campus Lübeck and the Research Center Borstel, Leibniz Lung Center, Borstel, Germany; 2 LungenClinic Großhansdorf, Großhansdorf, Germany; 3 Airway Research Center North (ARCN), Member of the German Center for Lung Research (DZL), Großhansdorf, Germany; 4 Institute of Human Genetics, Christian-Albrechts-University Kiel and University Medical Center Schleswig-Holstein, Campus Kiel, Kiel, Germany; 5 Institute of Human Genetics, Ulm University and University Medical Center Ulm, Ulm, Germany

**Keywords:** Survivin (BIRC5), Regucalcin (RGN), Transcriptome, Methylome, Non-small cell lung cancer (NSCLC), Proliferation, Senescence

## Abstract

Recently, it was shown that the epigenetic age of non-small cell lung cancer (NSCLC) tissues is different from the chronological age of patients. Here, we demonstrate that Regucalcin and Survivin, molecules which are known to be involved in the process of aging and overcoming aging, are epigenetically modified in NSCLC tissues compared to corresponding tumor-free tissues from the same donors by using methylome bead chip and corresponding transcriptome analyses. A high expression of Survivin on the RNA level was negatively correlated with patients’ survival in adenocarcinomas while a high Regucalcin expression was correlated positively. In stage 1 adenocarcinomas, this separation is even sharper for both genes. Within these, adenocarcinomas, smokers with low expression of Survivin show a better outcome, while the high expression of Regucalcin seems to be protective in never smokers. On the protein level, these molecules were detected by immunohistochemistry using tissue microarrays. Since Survivin can be secreted and we observed a high abundance of the protein also in the adjacent immune cells of the tumor microenvironment, an effect on benign cells can be assumed. These findings show that epigenetic re-programming of Survivin and Regucalcin in non-small cell lung cancer leads to enhanced expression of Survivin and reduced expression of Regucalcin, with a possible role of both molecules as predictive markers.

## Main text

The human life span is limited through a gradual loss of function which occurs in the organism at different levels. This process of aging leads to diseases such as osteoporosis, pulmonary insufficiency, and cancer [1]. Utilizing Horvath’s clock, we recently showed that different epigenetic aging takes place in NSCLC entities and does not match the chronological age of the patients [2]. Two genes known to be centrally involved in proliferation and senescence processes, which are not considered in Horvath’s algorithm, are Survivin (*BIRC5*) and Regucalcin (*RGN*). *BIRC5* is known as a dual functional regulator that inhibits apoptosis and promotes proliferation with a negative effect on survival [3, 4], while *RGN* is associated with aging and positively contributes to the outcome of cancer patients [5].

**Fig. 1:**
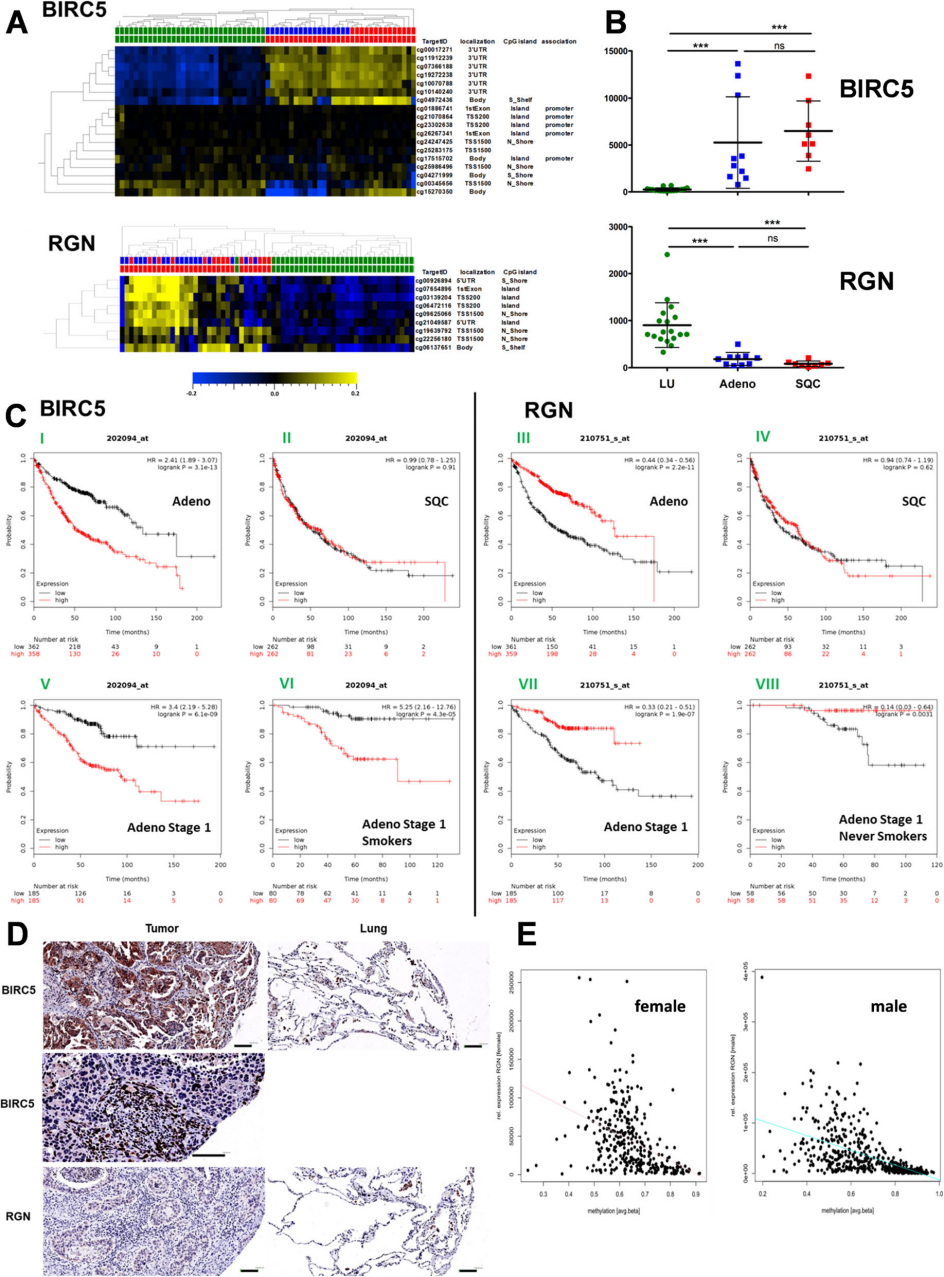
BIRC5 and RGN are differentially methylated and expressed in NSCLC and tumor-free lung tissues. **a** Normalized methylation analyses of CpG loci on Human Methylation 450k BeadChip (upper top bar: green—controls, blue—adenocarcinomas, red—squamous cell carcinomas; lower top bar: green—controls, red—tumor samples; heatmap: yellow high, blue low DNA methylation values; mean DNA methylation = 0; the target IDs of the CpG loci, their localization, and functional association are indicated). **b** Relative gene expression level of *BIRC5* and *RGN* as quantile-normalized expression values of tumor-free lung tissues and matched tumors depicting the mean in the 99.9% confidence interval with error bars (green: controls, blue: adenocarcinomas, red: squamous cell carcinomas). One-way ANOVA and Tukey’s multiple comparison test were used with *p* ≤ 0.001 (***) regarded significant. **c** Survival analysis using RNA expression data retrieved from kmplot.com selecting Affymetrix-ID 202094 for *BIRC5* and 210751_s for *RGN*. **d** Exemplary results from IHC analyses displaying expression of BIRC5 (upper and middle panel) and RGN (lower panel) on the protein level in tumors (left) and tumor-free lung tissues (right) from the same donors (scale bars = 100 μm). Middle panel shows the expression of BIRC5 in tumor-adjacent immune cells. **e** Correlation of DNA methylation (cg06472116, located on chr. X) vs. gene expression (*RGN*) in female [left graph] (Pearson’s correlation coef − 0.4127; *p* value < 1.1550 × 10^–15^) and male [right graph] donors (Pearson’s correlation coef − 0.5682; *p* value < 1.3136 × 10^–44^)

Therefore, we investigated epigenetic modifications of the *BIRC5* and *RGN* genes in 33 patients (20 males, 13 females) by Infinium HumanMethylation450k BeadChips (Illumia Inc.) as previously described. All tumors were primary NSCLC and comprised 15 adenocarcinomas and 18 squamous cell carcinomas of the lung; the mean patient age at surgery was 65.6 years. Of these 33 patients, 18 were analyzed by transcriptome analysis as recently shown [6]. Relative quantile-normalized gene expression values for *BIRC5* and *RGN* were extracted from the GEO-dataset GSE74706 and analyzed with GraphPad Prism v.6 (Fig. 1b). Protein level expression was determined by immunohistochemistry (IHC) utilizing polyclonal antibodies (BIRC5: Thermo Fischer Scientific, 1:50 dilution; RGN: Antibodies-online.com, 1:100 dilution) and ZytoChem-Plus-HRP with aminoethylcarbazole as a chromogen (Zytomed Systems) on tissue microarrays of formalin-fixed NSCLC tissues from 40 cases (21 adenocarcinomas, 19 squamous cell carcinomas) and matched controls (Fig. 1d). Hierarchical clustering of methylation levels of *BIRC5* and *RGN* CpG loci obtained by GenomeStudio software was visualized by OMICS Explorer 2.1 (Qlucore). For validating the outcome of the DNA methylation data analysis, additional methylation data of 919 cases (412 squamous cell carcinomas and 507 adenocarcinomas, including controls) provided by the TCGA consortium was accessed from the GDC data portal (https://portal.gdc.cancer.gov/).

Regarding the methylation of *BIRC5* and *RGN*, we observed a clear differentiation of tumor-free and lung cancer tissues (Fig. 1a). Loci located in the downstream region of the *BIRC5* gene (3′UTR, gene body; according to the information provided by the manufacturer) were hypermethylated in the tumor samples as compared to the controls whereas loci located in the transcription start site (TSS) and CpG islands were not aberrantly methylated in our analysis. In contrast, CpG loci of the *RGN* gene located in CpG islands and at the transcription start site were hypermethylated in tumor samples. This outcome is in line [7] with an unambiguous increase in RNA transcription levels of *BIRC5* in tumors as compared to corresponding tumor-free tissues and clearly decreased transcription levels of *RGN* (Fig. 1b). In tumor samples, DNA hypermethylation of the 6 CpG loci located in the 3′UTR of *BIRC5* correlated significantly with an increase in BIRC5 expression (Pearson correlation coefficient (coeff) 0.515 to 0.633, *p* value 0.0013 to 3.442 × 10^−5^), while hypermethylation of loci located in the TSS200 and 5′UTR of *RGN* correlated inversely with the expression of *RGN* (cg03139204 coeff − 0.373, *p* value 0.0249; cg06472116 coeff − 0.363, *p* value 0.0298; cg19639792 coeff − 0.454, *p* value 0.0054). These findings were validated in the TCGA cohort (BIRC5: coeff (median) 0.222 (0.070–0.273), *p* value (median) 6.1 × 10^−10^ (0.042–5.6 × 10^−16^); RGN (female): coeff − 0.409 to − 0.375, *p* value 4.15 × 10^−15^–9.14 × 10^−13^; RGN (male): coeff − 0.573 to − 0.507, *p* value 1.63 × 10^−43^–7.04 × 10^−33^). For *RGN*, we show a clear negative correlation of gene transcription and methylation in the promoter site, which points towards an aberrant methylation in NSCLC, exemplarily visualized in Fig. 1e. While no significant differences in BIRC5 expression between adenocarcinomas and squamous cell carcinomas have been detected (*p* = 0.53, *t* test), adenocarcinomas showed by trend a slightly higher *RGN* RNA level than squamous cell carcinomas (*p* = 0.053, *t* test) (Fig. 1b). This finding is supported by analyzing gene expression data of a larger lung cancer dataset provided by the TCGA consortium (*p* < 2.2 × 10^−16^, *t* test). The hypermethylation of *BIRC5* taking place mainly in the 3′-UTR region showing no differences at the promoter region suggests a secondary regulation, e.g., by a RNA or LncRNA. The methylation differences of *BIRC5* therefore seem to be non-aberrant and dependent possibly on the cell of origin, which is reflected in the separation of adenocarcinomas and squamous cell carcinomas by analyzing the methylation of *BIRC5* (Fig. 1a).

Kaplan-Meier analyses were performed in a separate collective of patients from data generated using Affymetrix chips and retrieved from kmplot.com (Fig. 1c) [8]. A high expression of *BIRC5* on the RNA level was negatively correlated with the survival of patients in adenocarcinomas (Fig. 1c (I)) while a high *RGN* expression was positively correlated with survival (Fig. 1c (III)). No such correlation was found in squamous cell carcinomas (Fig. 1c (II and IV)). This separation was even more distinct for both genes in stage 1 adenocarcinomas (Fig. 1c (V and VII)). Further inclusion of smoking habits revealed that smokers with low expression of *BIRC5* show a better outcome in stage 1 tumors (Fig. 1c (VI)), while high expression of *RGN* in stage 1 adenocarcinomas seems to be protective in never smokers (Fig. 1c (VIII)). Furthermore, we found similar data on the expression level differences between tumors and tumor-free tissues also in kmplot.com (*N* = 2437 tumors, 86 tumor-free. BIRC5: median tumors = 244, median tumor-free = 149 [*N* = 86]; RGN: median tumors = 192, median tumor-free = 383).

On the protein level, we observed an elevated expression of BIRC5 (Fig. 1d) in lung tumors, regardless of the histological sub-entity (adenocarcinoma/squamous cell carcinoma). Moreover, we frequently observed high levels of BIRC5 expression in tumor-adjacent immune cells (29/40 cases).

Interestingly, we found similar expression levels for RGN in the tumor and tumor-free areas, which are below the levels of BIRC5, suggesting regulation on the translational level or protein turnover (Fig. 1d).

In summary, we show that *BIRC5* and *RGN* are epigenetically modified in human NSCLC tissues compared to tumor-free tissues, resulting in increased (*BIRC5*) or decreased (*RGN*) transcription with a strong increase of BIRC5, but not a decrease of RGN on the protein level. We show correlations of both antithetic molecules with the survival of adenocarcinoma patients, especially in stage 1 and depending on smoking habits, which, although deserving further validation, might be useful in prognostic approaches.

## Data Availability

The datasets used and/or analyzed during the current study are available from the corresponding author on reasonable request.

## Abbreviations

BIRC5: Survivin
IHC: Immunohistochemistry
NSCLC: Non-small cell lung cancer
RGN: Regucalcin
TSS: Transcription start site
